# Trichodysplasia Spinulosa Polyomavirus Endothelial Infection, California, USA 

**DOI:** 10.3201/eid2809.220856

**Published:** 2022-09

**Authors:** Lauren Lawrence, Aihui Wang, Gregory Charville, Angus Toland, Benjamin Pinsky, Yasodha Natkunam, Sheren Younes, Henning Stehr, Dita Gratzinger

**Affiliations:** Stanford Healthcare, Palo Alto, California, USA (L. Lawrence, A. Toland);; Stanford University School of Medicine, Stanford, California, USA (A. Wang, G. Charville, B. Pinsky, Y. Natkunam, S. Younes, H. Stehr, D. Gratzinger)

**Keywords:** trichodysplasia spinulosa polyomavirus, viruses, TSPyV, trichodysplasia spinulosa, alphapolyomavirus, endotheliitis, California, United States

## Abstract

We describe 3 patients in California, USA, with trichodysplasia spinulosa polyomavirus (TSPyV) infection of endothelium after steroid administration. We detected TSPyV RNA in tissue specimens by in situ hybridization, which revealed localization to endothelial cells. These cases suggest that diseases associated with endothelial inflammation could be associated with TSPyV infection.

Trichodysplasia spinulosa polyomavirus (TSPyV) is an alphapolyomavirus whose primary clinical manifestation in a posttransplant setting is folliculocentric papular cutaneous eruptions, typically involving the face (*1*). Identification of TSPyV nucleic acids in tonsillar tissue has led to the speculation that lymphoid tissue might be a latency site (*2*); however, some disagreement exists in the literature as to whether the clinical diagnosis of trichodysplasia spinulosa reflects primary infection or reactivation of latent virus (*3*,*4*). Although cutaneous disease is the primary clinical manifestation of infection, TSPyV has been identified in blood, urine, cerebrospinal fluid, tonsils, and respiratory specimens by various methods, including nucleic acid detection, immunohistochemistry, and electron microscopy (*2*,*4*–*6*). TSPyV DNA loads can be high, especially in blood (up to 10^8^ viral copies/mL), months before the appearance of typical trichodysplasia spinulosa skin lesions (*4*).

This case study was part of a larger project approved by the Stanford Institutional Review Board (approval no. 58311) designed to explore oncogenesis by alphapolyomaviruses. We identified rare cases on our next generation sequencing panel of solid tumors with off-target, high quality reads that aligned to the TSPyV genome. We hypothesized that some of these cases might represent TSPyV-mediated neoplasms. Our cases comprised 1 patient with metastatic lung adenocarcinoma involving the brain (case 1), 1 patient with meningioma (case 2), and 1 patient with a metastatic perivascular epithelial cell tumor involving the liver (Table). It is unclear whether the mental status changes observed in cases 1 and 2 were attributable to viral infection or were secondary to the tumors (Table). All 3 patients received steroids immediately preceding resection. We performed in situ hybridization using a custom RNAScope probe that targeted the complete TSPyV viral genome (GenBank accession no. NC_014361.1) and RNAscope 2.5 HD Reagent Kit-RED (Advanced Cell Diagnostics, https://acdbio.com) to detect TSPyV RNA in cutaneous biopsy specimens. One case of cutaneous trichodysplasia spinulosa was used as a positive control for TSPyV RNA. We used the RNAscope 2.5 LS Probe-Hs-PPIB-sense probe (Advanced Cell Diagnostics), which is specific for peptidylprolyl isomerase B, to demonstrate the presence of intact RNA in the tissue sections. For negative in situ hybridization controls, we used tissue specimens from patients with Merkel cell polyomavirus-positive Merkel cell carcinoma and BK polyomavirus nephropathy and a T-cell lymphoma tissue microarray that included 1 case of Merkel cell polyomavirus-positive T-cell lymphoma.

**Table:** Case summaries of 3 patients with trichodysplasia spinulosa polyomavirus endothelial infection, California, USA*

Case no.	Histopathologic diagnosis	Immunosuppression	Localization of RNA	Clinical manifestation
1	Metastatic pulmonary adenocarcinoma involving brain	Dexamethasone (4 mg) leading up to resection; rituximab/bendamustine treatment completed 18 mo before resection for follicular lymphoma	Endothelium	Ground level fall, confusion, forgetfulness, ambulatory instability
2	Anaplastic meningioma	Dexamethasone (2 mg × 10 d, then 1 mg × 2 d) leading up to resection	Endothelium	Confusion, headaches, cheek numbness, ambulatory instability
3	Metastatic perivascular epithelial cell tumor involving liver	Gemcitabine/docetaxel completed 1 mo before resection; prednisone (8 mg orally 2×/d) for 1 mo leading up to resection	Endothelium	Abdominal pain, fever, vomiting, hypoxia

In contrast to our hypothesis, we found that TSPyV RNA did not localize to neoplastic cells. However, TSPyV RNA localized to the endothelium in all 3 cases (Figure). The BK polyomavirus nephropathy sections showed weak, orange-red discoloration of tubular epithelium (Figure, panel L) that was distinguishable from the bright red, granular staining patterns for TSPyV RNA observed in the positive control and tissue specimens from the 3 patients. This discoloration was interpreted as negative for TSPyV RNA by board-certified pathologists who had experience evaluating RNAScope in situ hybridizations. 

**Figure:**
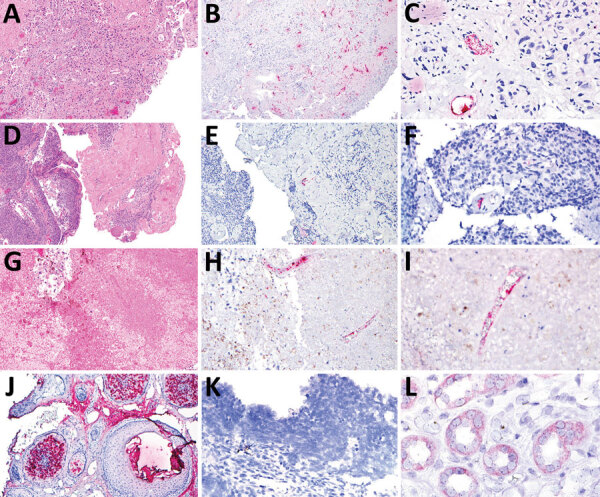
Tissue samples from 3 patients with trichodysplasia spinulosa polyomavirus endothelial infection, California, USA. We performed hematoxylin and eosin (H&E) staining and RNAScope in situ hybridization (ISH) to detect trichodysplasia spinulosa polyomavirus (TSPyV) in formalin-fixed, paraffin-embedded tissue specimens. Bright red, granular staining in endothelium indicates TSPyV RNA. A) Case 1, H&E staining, original magnification ×10; B) case 1, TSPyV ISH, original magnification ×10; C) case 1, TSPyV ISH, original magnification ×40; D) case 2, H&E staining, original magnification ×5; E) case 2, TSPyV ISH, original magnification ×10; F) case 2, TSPyV ISH, original magnification ×40; G) case 3, H&E staining, original magnification ×5; H) case 3, TSPyV ISH, original magnification ×10; I) case 3, TSPyV ISH, original magnification ×40; J) biopsy from patient with cutaneous trichodysplasia spinulosa (positive control), TSPyV ISH, original magnification ×10; K) cutaneous biopsy from patient with Merkel cell polyomavirus-positive Merkel cell carcinoma (negative control), TSPyV ISH, original magnification ×40; L) renal biopsy from patient with BK polyomavirus nephropathy (negative control), TSPyV ISH, original magnification ×60.

TSPyV is a DNA virus, and detection of TSPyV RNA indicates active viral replication and infection. Our in situ hybridization results suggest that TSPyV can infect endothelial cells, likely within various tissues. These cases provide insight into a potential cellular reservoir for TSPyV infection. In addition, these data raise the possibility that other diseases associated with endothelial inflammation could be associated with TSPyV infection. Overall, this small case series improves our knowledge of the scope of human TSPyV infection.
